# Contribution of Fcγ Receptor-Mediated Immunity to the Pathogenesis Caused by the Human Respiratory Syncytial Virus

**DOI:** 10.3389/fcimb.2019.00075

**Published:** 2019-03-29

**Authors:** Orlando A. Acevedo, Fabián E. Díaz, Tomas E. Beals, Felipe M. Benavente, Jorge A. Soto, Jorge Escobar-Vera, Pablo A. González, Alexis M. Kalergis

**Affiliations:** ^1^Millennium Institute on Immunology and Immunotherapy, Departamento de Genética Molecular y Microbiología, Facultad de Ciencias Biológicas, Pontificia Universidad Católica de Chile, Santiago, Chile; ^2^Laboratorio de Genética, Departamento Biomédico, Facultad de Ciencias de la Salud, Universidad de Antofagasta, Antofagasta, Chile; ^3^Departamento de Endocrinología, Facultad de Medicina, Pontificia Universidad Católica de Chile, Santiago, Chile

**Keywords:** hRSV, Fc gamma receptors, re-infection, inflammatory response, lung disease, immune complexes, opsonized virus

## Abstract

The human Respiratory Syncytial Virus (hRSV) is the leading cause of severe acute lower respiratory tract infections (ALRTIs) in humans at all ages and is the main cause of hospitalization due to pneumonia, asthma, and bronchiolitis in infants. hRSV symptoms mainly develop due to an excessive host immune and inflammatory response in the respiratory tissue. hRSV infection during life is frequent and likely because of non-optimal immunological memory is developed against this virus. Vaccine development against this pathogen has been delayed after the detrimental effects produced in children by vaccination with a formalin-inactivated hRSV preparation (FI-hRSV), which caused enhanced disease upon natural viral infection. Since then, several studies have focused on understanding the mechanisms underlying such disease exacerbation. Along these lines, several studies have suggested that antibodies elicited by immunization with FI-hRSV show low neutralizing capacity and promote the formation of immune complexes containing hRSV (hRSV-ICs), which contribute to hRSV pathogenesis through the engagement of Fc gamma receptors (FcγRs) expressed on the surface of immune cells. Furthermore, a role for FcγRs is supported by studies evaluating the contribution of these molecules to hRSV-induced disease. These studies have shown that FcγRs can modulate viral clearance by the host and the inflammatory response triggered by hRSV infection. In addition, ICs can facilitate viral entry into host cells expressing FcγRs, thus extending hRSV infectivity. In this article, we discuss current knowledge relative to the contribution of hRSV-ICs and FcγRs to the pathogenesis caused by hRSV and their putative role in the exacerbation of the disease caused by this virus after FI-hRSV vaccination. A better understanding FcγRs involvement in the immune response against hRSV will contribute to the development of new prophylactic or therapeutic tools to promote virus clearance with limited inflammatory damage to the airways.

## Introduction

The Human Respiratory Syncytial Virus (hRSV) is a single-stranded RNA enveloped virus belonging to the *Pneumoviridae* family (Amarasinghe et al., 2018). The viral particle has a filamentous structure, which consists in a nucleocapsid surrounded by a lipid bilayer envelope obtained from the plasma membrane of the host cell (El Omari et al., 2011). Importantly, infection by hRSV is the most frequent cause of severe acute lower respiratory tract infections (ALRTIs) in children younger than 5 years old (Scheltema et al., 2017) and infection during the first year of life is the main cause of hospitalization in infants (Song et al., 2016). According to epidemiological studies, during the past decade, nearly 33 million cases of new ALRTIs episodes affect children during the first months of life are due to hRSV infection each year (Shi et al., 2017). Therefore, infection by this virus represents a major health and socio-economic burden worldwide (Diez-Domingo et al., 2014; Amand et al., 2018).

Clinical manifestations caused by hRSV infection range from mild symptoms, such as rhinitis, to more severe consequences, which include bronchiolitis, and pneumonia (Pickles and DeVincenzo, 2015). Besides, extra-pulmonary manifestations of hRSV infection have also been reported to occur, such as acute neurological symptoms with seizures and ataxia observed in hRSV-infected children (Eisenhut, 2006; Bohmwald et al., 2015) and long-term behavioral and cognitive impairments in animal models (Espinoza et al., 2013).

Remarkably, it is known that most children become infected with hRSV during the first 2 years of life (Domachowske and Rosenberg, 1999), likely because hRSV can efficiently pass on from one individual to another, but also because of the capacity of this virus to negatively modulate both, T cell and B cell responses upon infection allowing frequent re-infections (PrabhuDas et al., 2011; Cespedes et al., 2014; Zhivaki et al., 2017). These features are thought to be mediated by host and viral factors. For instance, it is known that infants show reduced capacity to produce neutralizing antibodies against hRSV, as compared to adults making the former more susceptible to recurrent infections (Siegrist and Aspinall, 2009). Although maternally-delivered antibodies (matAbs) are reported to delay the onset of primary hRSV infection, their presence in the blood of infants is not associated with the development of less severe disease symptoms (Jans et al., 2017). These observations suggest that antibody-mediated neutralization of hRSV may not be sufficient by itself to limit hRSV infection and disease severity. Furthermore, hRSV encodes several proteins that have the ability to negatively modulate or impair the host antiviral immune response, therefore contributing to re-infections (Mason et al., 2003; Cespedes et al., 2014; Saint et al., 2015; Bohmwald et al., 2016; Gomez et al., 2016; Canedo-Marroquin et al., 2017; Ward et al., 2017). Such knowledge is relevant for designing novel vaccines and therapeutic approaches that can prevent the pathology caused by hRSV. As a result, several clinical trials are currently in progress to assess the safety and effectiveness of different hRSV vaccine candidates (Cautivo et al., 2010; Rey-Jurado and Kalergis, 2017; Rezaee et al., 2017). Among them, we have developed a unique approach to be administered to newborns and young infants. Immunization in the mouse model with a recombinant bacillus of Calmette-Guérin (BCG) that expresses the nucleoprotein (N) of hRSV (rBCG-N-hRSV) induce the production of neutralizing antibodies against hRSV and a T helper 1 (Th1) cellular immunity that protects from hRSV associated-lung pathology by decreasing the infiltration of inflammatory immune cells into the lungs and reduce viral loads in the airways of hRSV-infected mice (Bueno et al., 2008; Cautivo et al., 2010; Leyrat et al., 2014) Furthermore, a single low dose of this vaccine produced using current good manufacturing practices (cGMP), conferred protection against hRSV infection in the mouse model (Cespedes et al., 2017). Given these results, this recombinant-based vaccine arises as a promising candidate to prevent lung damage caused by this virus (Cespedes et al., 2017). In this context, it is possible that a mechanism that contributes to the prevention of hRSV pathology following rBCG-N-hRSV vaccination is the induction of antibodies that recognize the hRSV N protein, which is necessary for viral replication and the inhibition of the immunological synapse (IS) between DCs and T cells that promote T- cell activation (Cespedes et al., 2014). Therefore, if the hRSV N protein becomes neutralized by antibodies during infection it cannot contribute to viral replication, but also will fail in its ability to impair the formation of the IS between DCs and T cells, thus hampering a proper immune response against hRSV. Furthermore, a recent publication from our group shows that immunization with rBCG-N-hRSV can induce the production of antibodies against other hRSV proteins, such as F and G which can serve to neutralize infection, therefore reducing hRSV associated pathology (Soto et al., 2018).

In contrast, vaccine candidates from other groups use the F protein as a target antigen to confer immunity. For example, Novavax Inc. is currently performing a clinical trial based on the use of nanoparticles linked with hRSV F protein to induce the production of neutralizing antibodies against hRSV (Mazur et al., 2018).

Similarly, Janssen is currently testing adenovirus based vector vaccines, encoding pre-fusion forms of the hRSV F protein that also induce the production of anti-hRSV neutralizing antibodies (Mazur et al., 2018).

Finally, other live attenuated vaccines as is the case of rBCG-N-hRSV are based in attenuated hRSV that lack some particular proteins such as M2-2, NS2, or both (Mazur et al., 2018).

Together, these data indicate that it is of vital importance to delineate the mechanisms contributing to hRSV induced pathology in order to prevent or treat infection.

At this latter point, recurrent hRSV re-infection episodes which are common thorough life have encouraged the generation of studies that seek to define the mechanisms responsible for what is considered an impaired or non-optimal immune response elicited against hRSV upon infection to account for re-infection episodes (Openshaw and Chiu, 2013; Cespedes et al., 2014; Shao et al., 2015). Along these lines, a role for the interaction between immune complexes consisting of IgGs and hRSV (ICs) with Fc gamma receptors (FcγRs) could be a process contributing to both, re-infection episodes, and enhancement of hRSV-disease elicited by vaccination with formalin-inactivated hRSV (FI-hRSV) and later hRSV natural infection (Kim et al., 1969). This hypothesis is supported by the fact that high amounts of antibodies with low neutralizing activity can be induced by immunization with FI-hRSV, which correlates with enhancement of the hRSV-induced disease (Kapikian et al., 1969; Kim et al., 1969). Therefore, it is possible that these low affinity antibodies promote the infection of FcγR-bearing cells through a phenomena called antibody dependent enhancement (ADE), as previously observed for other viruses (Yip et al., 2014; Gu et al., 2015; Flipse et al., 2016). Furthermore, *in vitro* and *in vivo* studies have shown that the blockade or absence of particular FcγRs expressed on the surface of immune cells can modulate the immune response against this virus and the onset of hRSV-induced disease (Osiowy et al., 1994; Kruijsen et al., 2013; Gomez et al., 2016; van Erp et al., 2018). In this article, we review and discuss the current understanding on the contribution of FcγRs to infection and the modulation of the immune response against hRSV both, *in vitro* and *in vivo* and their impact on hRSV-induced pathology.

### The Family of Fc Receptors for IgG (FcγRs)

Fc-gamma receptors (FcγRs) bind to immunoglobulin G (IgG) antibodies (Ab), by recognizing the Fc region of the IgG, which promotes receptor clustering on the cell surface and the phosphorylation of tyrosine residues present on signaling motifs within the intracellular region of these receptors. FcγRs engagement ultimately leads to signaling cascades in the cell that can result in the expression of surface molecules and secretion of soluble mediators to modulate the host immune responses (Getahun and Cambier, 2015; Renner et al., 2016); (Soto et al., 2018).

Importantly, these types of receptors are expressed on the surface of immune cells, such as neutrophils, dendritic cells (DCs) and macrophages, among others (Zhang et al., 2004). In general, classic members of this family of proteins were classified according to their immune-modulatory properties, which either promote or inhibit inflammatory responses (Nimmerjahn and Ravetch, 2008; Guilliams et al., 2014). However, FcγRs can also be classified as type-I or type-II, based on their capacity to interact with the two (open or closed) conformational states of the IgG Fc domain (Banegas Banegas et al., 1987). Type-I FcγRs include the classic FcγRs and can only be engaged by the IgG Fc domain in the open conformation state (Banegas Banegas et al., 1987). In contrast, type-II (non-canonical FcγRs), include C-type lectin receptors CD23 and Dendritic Cell-specific Intercellular Adhesion Molecule-3-Grabbing Non-integrin (DC-SIGN), which preferentially bind IgG Fcs in a closed conformation (Banegas Banegas et al., 1987).

In humans, the so-called classic FcγRs are known as: FcγRI (CD64), FcγRIIa (CD32a), FcγRIIb (CD32b), FcγRIIc (CD32c), FcγRIIIa (CD16a), and FcγRIIIb (CD16b) (Table 1) (Tripp et al., 2002; Guilliams et al., 2014). Among them, a study performed during 2002 indicates that the expression of FcγRIIIa is increased in Natural Killer cells (NK cells) from patients with severe hRSV associated pathology. Thus, suggesting that this receptor and this particular cell population could be contributing to hRSV disease (Table 2, Tripp et al., 2002). Nevertheless, there are two more non-classic human Fc-gamma receptors: neonatal Fc-receptor (FcRn) and cytosolic tripartite motif (TRIM) 21 that bind IgG once internalized into the cells (Guilliams et al., 2014). However, there is no study about it contribution to hRSV induced pathology in hRSV positive patients (Table 2). Importantly, all canonical FcγRs with the exception of FcγRIIb are involved in activating functions, such as phagocytosis, antibody-dependent cellular cytotoxicity (ADCC) and the release of inflammatory cytokines following FcγR-crosslinking by IgG-opsonized complexes (Guilliams et al., 2014). The activation of such processes relies on the Src-family kinase-mediated phosphorylation of an Immunoreceptor Tyrosine-based Activating Motif (ITAM) that is located in the cytoplasmic portion of these activating Fc-receptors (Nimmerjahn and Ravetch, 2008). Subsequently, phosphoinositide 3-kinase (PI3K) is activated, which generates phosphatidylinositol trisphosphates (PIP3s), leading to the recruitment of Bruton's tyrosine kinase (BTK) and the activation of phospholipase Cγ (PLCγ), which promotes the release of calcium (Ca^2+^) from the endoplasmic reticulum (ER) that in turn activates cell effector functions (Nimmerjahn and Ravetch, 2008).

**Table 1:** Classification of currently described human Fcγ Receptors, and evidences of their role in hRSV-induced pathogenesis.

**Type**	**Receptor**	**Alternative name/CD**	**Main function**	**Evidence after hRSV infection**	**Suggested role**	**References indicating a role during hRSV infection**
Classical FcγRs (Recognize ICs on the cell surface)	FcγRI	CD64	Activating	—^a^	—	—
	FcγRIIa	CD32a	Activating	—	—	—
	FcγRIIb	CD32b	Inhibitory	—	—	—
	FcγRIIc	CD32c	Activating	—	—	—
	FcγRIIIa	CD16a	Activating	Increased presence of FcγRIIIA^+^ NK cells, and lung damage in patients with severe hRSV infections	The expression of FcγRIIIA on NK cells negatively influences the immune response during hRSV infection	Tripp et al., 2002
	FcγRIIIb	CD16b	Activating	—	—	—
Non-classical FcγRs (C-type lectins that recognize ICs on cell surface or non-classic FcγRs that recognize ICs inside the cell)	CD23	CD23		—	—	—
	DC-SIGN	CD209	Recognition of glycans through a carbohydrate recognition domain (CRD)	*In vitro*: mAb-blockade of DC-SIGN increases human DC maturation markers (CD80, CD86) after hRSV infection.	hRSV-DC interaction through DC-SIGN might impair DC maturation	Johnson et al., 2012
	FcRn	—	Control of endosomal routing	—	—	—
	TRIM 21	—	Elimination of ICs via recruitment of the proteasomal machinery	—	—	—

a *No data are available*.

**Table 2:** Classification of currently described mouse Fcγ Receptors, and evidences of their role in hRSV-induced pathogenesis.

**Type**	**Receptor**	**Main function**	**Evidence after hRSV infection**	**Suggested role**	**References**
Classical FcγRs (Recognize ICs on the cell surface)	FcγRI	Activating	—	—	—
	FcγRIIb	Inhibitory	*In vivo*: FcγRIIb^−/−^ mice display increased lung neutrophil infiltration but decreased viral loads	Anti-inflammatory role	Gomez et al., 2016
			*In vitro*: WT mice-derived BMDCs loaded with hRSV-ICs were not able to induce the production of IL-2 by CD4^+^ T cells as compared with FcγRIII^−/−^ mice-derived BMDCs	The engagement of FcγRIII by hRSV-ICs impairs DC-mediated T cell activation	Gomez et al., 2016.
			*In vitro*: FcγRIIb^−/−^mice-derived BMDCs loaded with hRSV-ICs showed unaltered capacity to induce the secretion of IFNγ by CD4^+^ T cells	DC-mediated stimulation of IFN-γ secretion by CD4^+^ T cells does not depend on the presence of the inhibitory FcγRIIb	Kruijsen et al., 2010
	FcγRIII	Activating	*In vivo*: FcγRIII^−/−^ mice display decreased neutrophil recruitment and higher viral loads	Pro-inflammatory role, promotion of viral clearance	Gomez et al., 2016
			*In vitro*: FcγRIII^−/−^ mice-derived BMDCs loaded with hRSV-ICs showed restored capacity to induce the production of IL-2 by CD4^+^ T cells	The engagement of FcγRIII by hRSV-ICs impairs DC-mediated T cell activation	Gomez et al., 2016
	FcγRIV	Activating	—^b^	—	—
Non-classical FcγRs (FcγRs that recognize ICs inside the cell)	FcRn	IgG recycling	*In vitro: FcγRn*^−/−^ mice-derived BMDCs loaded with hRSV-IC display unaltered capacity to induce IFN-γ production by CD4+ T cells. *In vivo*: FcRn^−/−^ and WT mice display similar CD4^+^ IFN-γ production after hRSV-IC challenge	FcRn does not modulate DC-mediated CD4^+^ T cell activation	Kruijsen et al., 2013
	TRIM 21	Elimination of ICs via recruitment of the proteasomal machinery	—	—	—

b *No data are available*.

In contrast, FcγRIIb which is able to terminate the activation cascades associated with the engagement of activating FcγRs (Malbec et al., 1998), is also known as the inhibitory FcγR. During this process FcγRIIb becomes engaged by ICs and it co-aggregates with activating receptors. Following that, different recruited kinases phosphorylate a conserved tyrosine within an Immunoreceptor Tyrosine-based Inhibitory Motif (ITIM) located in the cytoplasmic tail of FcγRIIb (Malbec et al., 1998). This phosphorylation step leads to the recruitment of tyrosine phosphatases SHP-1 and SHP-2, as well as the inositol phosphatases SHIP-1 and SHIP-2 that suppress the activating signals derived from activating FcγRs (D'Ambrosio et al., 1995; Ono et al., 1996).

In the mouse, there are four different canonical FcγRs expressed on the cell surface: FcγRI, FcγRIIb, FcγRIII, and FcγRIV (Table 2) (Nimmerjahn and Ravetch, 2008). Among them, FcγRI, FcγRIII, and FcγRIV are activating, whereas FcγRIIb is the only one that is inhibitory. Of interest, a pro-inflammatory role for the FcγRIII receptor has been reported during hRSV infection in the mouse model (Gomez et al., 2016), whereas the inhibitory FcγRIIb has been shown to hamper inflammatory reactions during allergic-like rhinitis (Malbec et al., 1998), allergic asthma (D'Ambrosio et al., 1995), and hRSV infection (Gomez et al., 2016). Therefore, such receptors appear as attractive targets for novel therapeutic approaches against this kind of diseases.

### Contribution of FcγRs to Neutrophil Recruitment, Viral Replication, and Lung Damage During hRSV-Induced Pathology

Based on animal studies, neutrophils have been described to promote inflammation and tissue damage during hRSV infection (Yasui et al., 2005). In addition, other studies in mice that evaluated the role of FcγRs on the lung damage produced by neutrophils in models of acute lung injury (ALI), which resembles those caused by hRSV infection (Zhang et al., 2016), have suggested that animals lacking activating FcγRs (FcRγ^−/−^ mice) can be protected from ALI triggered by administration of IgG mAbs that recognize self-antigens, such as MHC-I molecules (Looney et al., 2006). Supporting a role for neutrophils and activating FcγRs in this model of lung injury, the same study showed that ALI was observed when *FcR*γ^−/−^ mice were adoptively transferred with wild-type neutrophils followed by the administration anti-MHC-I mAbs (Looney et al., 2006). Taken together, these results suggest that lung disease in this model is dependent on the expression of activating FcγRs by neutrophils.

**Figure 1:**
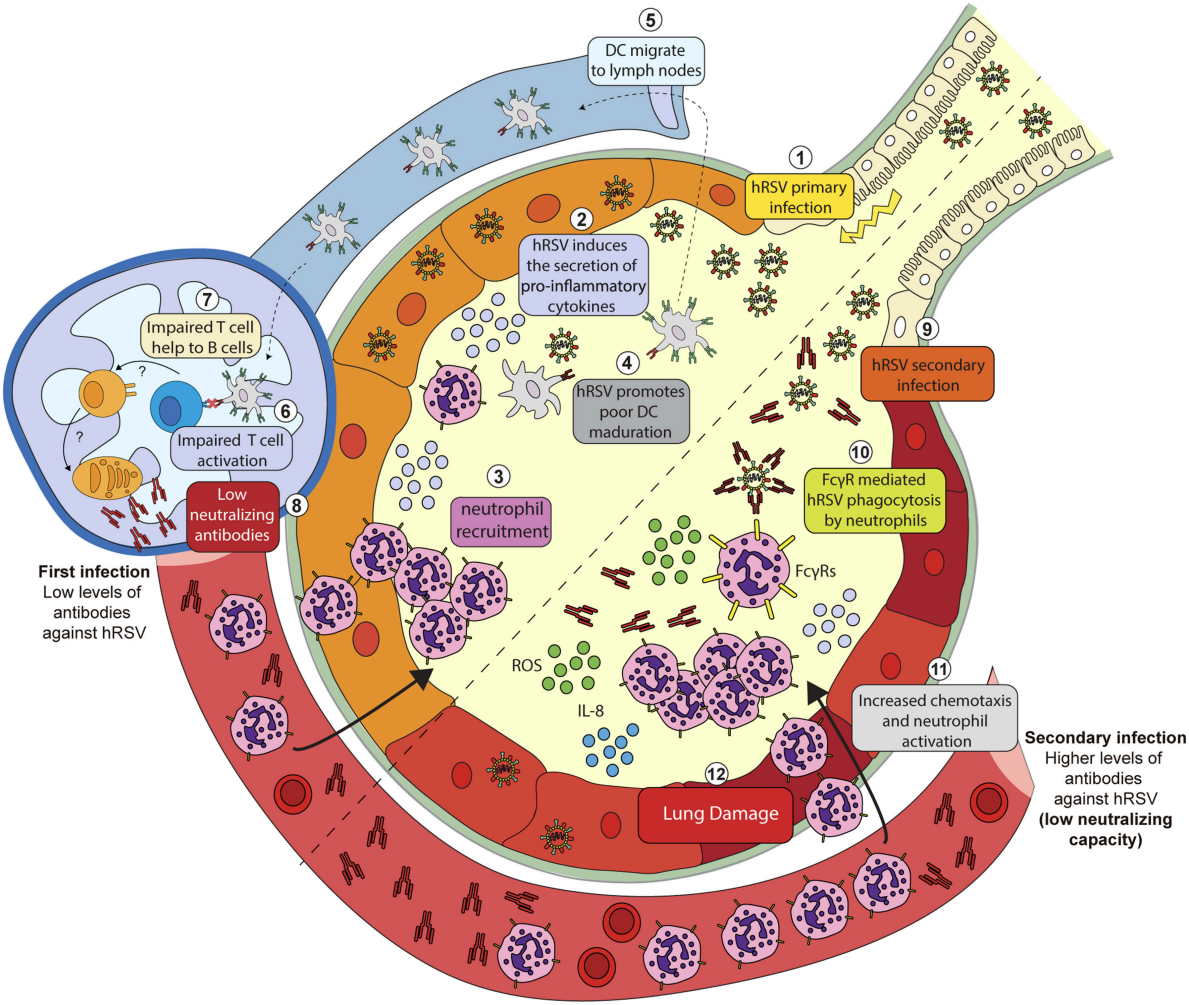
Putative mechanisms explaining hRSV-induced inflammation due to hRSV-IC interaction with Fc-gamma receptors expressed on the surface of neutrophils. During a primary infection (1) hRSV induces the secretion of pro-inflammatory cytokines (2) and chemokines that promote neutrophil recruitment to the lungs and the airways (3). During infection, hRSV is phagocyted by DCs and impair its maturation (4). Infected DCs migrate to lymph nodes (5) but fail to activate T cells (6). By a poorly understood mechanism, T cells fail to help naïve B cells (7) and promote the proliferation of plasma cells that produce anti-hRSV antibodies with a low neutralizing capacity (8). Serum antibodies produced after a primary hRSV infection can opsonize hRSV during secondary infection (9). Opsonized hRSV is then phagocyted by neutrophils through FcγRs (10). The infection of these cells triggers the release of cytokines such as IL-8 that promote the activation and the recruitment of neutrophils (11). Activated neutrophils then release metabolic products, i.e., reactive oxygen species (ROS) that promote lung damage and inflammation (12).

For the case of hRSV infection, it has been shown that the recruitment of neutrophils to the lungs of infected mice is modulated by the presence of different FcγRs (Figure 1) (Gomez et al., 2016). For instance, it was reported that animals lacking the activating FcγRIII (FcγRIII^−/−^) showed decreased neutrophil recruitment and higher viral loads (Gomez et al., 2016), suggesting that FcγRIII could play a pro-inflammatory role during hRSV primary infection and promotes viral clearance. Consistent with the results described above, mice lacking the inhibitory FcγRIIb (FcγRIIb^−/−^) showed increased neutrophil infiltration in lungs due to hRSV infection but decreased viral loads (Gomez et al., 2016), thus suggesting that this receptor can play an anti-inflammatory role during hRSV-induced disease despite it contributes to viral replication (Gomez et al., 2016).

An *in vitro* study using human neutrophils showed that hRSV-ICs, established with hRSV and anti-hRSV autologous serum, but not free hRSV or antibodies alone, could promote the release of reactive oxygen species (ROS) by neutrophils, which could contribute to lung tissue damage (Figure 1) (Kaul et al., 1981; Winterbourn et al., 2016). Therefore, it is possible that the activation of neutrophils, mediated by the engagement of FcγRs likely occurs under physiological conditions, when individuals become infected. This notion, is further supported by a study showing increased release of IL-8 by human neutrophils challenged with opsonized hRSV (Arnold et al., 1994). This cytokine is relevant, as it has been described that secreted IL-8 works as a chemotactic signal for neutrophils that induces their activation leading to pro-inflammatory responses (Henkels et al., 2011). This *in vitro* evidence suggests that the engagement of FcγRs can activate neutrophils and therefore contribute to lung inflammation and the progression of hRSV disease (Figure 1).

### Modulation of Dendritic Cell Function by FcγRs and ICs Containing hRSV: Implications for T Cell Immunity

Dendritic cells (DCs) can modulate the immune response during viral infections after capturing ICs through either, activating or inhibitory FcγRs (Guilliams et al., 2014). Along these lines, IgG–antigen complexes can trigger activating signals in human DCs (hDCs) after binding to FcγRIII and promote an inflammatory response (Bandukwala et al., 2007). In contrast, binding of ICs to the inhibitory FcγRIIb trigger inhibiting signals that can lead to reduced inflammation (Boruchov et al., 2005). In addition, it has been described that hRSV-ICs containing either, neutralizing or non-neutralizing antibodies can modulate DC function and subsequent T cell responses elicited by the antigen presentation of these cells (Kruijsen et al., 2013; Gomez et al., 2016).

In the context of hRSV infection, it has been reported that DC-mediated T cell activation and IFN-γ production by these cells, is modulated by the presence of activating FcγRs on the DC surface (Kruijsen et al., 2013). In this case, it was observed that DCs derived from WT adult mice were able to induce the production of IFN-γ by CD4^+^ T cells in the presence of anti-hRSV immune serum obtained from mice being challenged with hRSV (Kruijsen et al., 2013). Nevertheless, this observed increase in the IFN-γ response by CD4^+^ T cells was reduced when the DCs were derived from FcRγ^−/−^ mice. Therefore, the expression of all activating FcγRs on the DC surface is required to promote the production of this cytokine by CD4^+^ T cells. Remarkably, unaltered secretion of IFN-γ by CD4^+^ T cells was observed in DCs derived from FcγRIIb^−/−^ mice, when compared to WT mice (Kruijsen et al., 2013), indicating that DC-mediated stimulation of IFN-γ secretion by CD4^+^ T cells does not depend on the presence of the inhibitory FcγRIIb (Kruijsen et al., 2013).

Interestingly, another report indicates that CD4^+^ T cells represent an important source of IFN-γ during neonatal hRSV infection in the murine model, which is required to prevent re-infection and disease severity in adult mice (Lee et al., 2008). Thus, it is possible that activating FcγRs contribute to prevent re-infection during adulthood, by promoting IFN-γ production by CD4^+^ T cells through DC-mediated antigen presentation. However, it is necessary to determine whether activating FcγRs on the DC surface also modulate the production of this cytokine by neonatal CD4^+^ T-cells to prevent re-infection.

Recent studies have shown that another IgG Fc receptor, particularly the neonatal Fc receptor for IgG (FcRn), which is a non-classical Fc receptor that binds IgG at acidic pH (<6,5) (Qiao et al., 2008), does not contribute to the activation of CD4^+^ T cells when DCs are loaded with hRSV-ICs (Kruijsen et al., 2013). Moreover, Bone Marrow-Derived DCs (BMDCs) from FcγRn^−/−^ mice exhibit unaltered capacity to induce the production of IFN-γ by CD4^+^ T-cells (Kruijsen et al., 2013). These results were validated *in vivo*, as FcγRn^−/−^ mice also displayed unaltered IFN-γ production by CD4^+^ T-cells after being intranasally challenged with hRSV-ICs (Kruijsen et al., 2013).

Results from our group indicate that BMDCs display a reduced capacity to induce IL-2 production by CD4^+^ T cells after being loaded with hRSV-ICs that had the neutralizing antibody Palivizumab (Synagis^TM^) (Gomez et al., 2016). In contrast, when the assay was performed with BMDCs derived from either, FcγRIII^−/−^ or FcγRIIb^−/−^ mice IL-2 secretion by CD4^+^ T cells was restored. This results prompts that when present, these receptors impair the capacity of DCs to induce the secretion of IL-2 by CD4^+^ T cells. It should be noted that, the production of this cytokine is required for the generation of memory regulatory CD4^+^ T cells (Tregs), which perform anti-inflammatory functions during hRSV infection and protect against re-infections (Durant et al., 2013). Thus, it is possible that both, FcγRIII and FcγRIIb contribute to hRSV pathogenesis and re-infection by impairing the capacity of DCs to promote the production of IL-2 by CD4^+^ T cells.

### Type II FcγRs Expressed on the Surface of Human DCs Contribute to Immune Responses Against hRSV

In humans, the presence of two types of FcγRs has been recognized (Banegas Banegas et al., 1987). Type-I FcγRs are members of the immunoglobulin superfamily and can be either activating or inhibitory (Nimmerjahn and Ravetch, 2005, 2008). In contrast, Type-II FcγRs are members of the C-type lectin receptor family and comprise two different members: the IgE receptor and the surface protein DC-SIGN (Banegas Banegas et al., 1987; Miettinen, 2004), which is able to recognize the Fc portion of IgG (Kaneko et al., 2006; Svajger et al., 2010), but also the G protein expressed by hRSV (Johnson et al., 2012). Of interest, studies evaluating the role of DC-SIGN in hDCs during hRSV infection, showed that the blockade of this receptor with specific mAbs led to an increase in the expression of maturation markers, such as CD80 and CD86 following hRSV infection (Johnson et al., 2012). This suggests that the interaction between hRSV surface proteins and DC-SIGN can suppress some aspects of DC activation in humans, thus contributing to an impaired protective immunity following hRSV infection. However, further studies are required to study the influence of this receptor during infection *in vivo* and hRSV-induced pathology, as well as its consequences on DC mediated T-cell activation.

### Contribution of ADE to hRSV Re-infection Episodes

In a recent study, it was shown that young infants (i.e., <3 months old) generate a highly neutralizing antibody response that is biased from the post-fusion to the pre-fusion form of hRSV F protein. However, as children become older (i.e., from <3 months old to >6 months old), this response is re-directed against post-fusion conformation antigens (Goodwin et al., 2018). Thus, the antibodies generated display a weak neutralizing capacity that fail to prevent hRSV infection. Therefore, it is possible that the generation of a pool of low-neutralizing antibodies during infancy can facilitate infection of immune cells that express FcγRs, a phenomenon called ADE of infection that has been observed for other viruses such as dengue virus (Flipse et al., 2016), acute respiratory syndrome coronavirus (Yip et al., 2014) and porcine reproductive and respiratory syndrome virus infection (Gu et al., 2015). In this context, antibodies might exert different effector functions through their Fc regions and for hRSV, ADE during infection is an effect that has been reported in *in vitro* studies (Gimenez et al., 1989; Krilov et al., 1989; Osiowy et al., 1994). However, a role of ADE during hRSV pathogenesis *in vivo* has been proposed, but remains to be confirmed during re-infection.

To date, *in vitro* enhancement of infection of monocyte-derived cell lines due by FcγR binding by mAbs and patient sera has been reported (Gimenez et al., 1989; Krilov et al., 1989; Osiowy et al., 1994), demonstrating that non-neutralizing mAbs can enhance the infection of phagocytic cell lines expressing these receptors (Gimenez et al., 1996). Further, when neutralizing antibodies were applied at sub-neutralizing concentrations (i.e., diluted), they induced ADE in phagocytic cells bearing FcγRs. This was also observed using human sera and purified human immunoglobulin (IVIg) (van Erp et al., 2017). Together, these results suggest that the interaction of hRSV-ICs generated with low neutralizing antibodies can promote the infection of immune cells *in vitro*, therefore contributing to hRSV pathogenesis under physiological conditions.

### Contribution of ICs Containing hRSV to Enhanced Disease Elicited by Vaccination With Formalin-Inactivated hRSV

**Figure 2:**
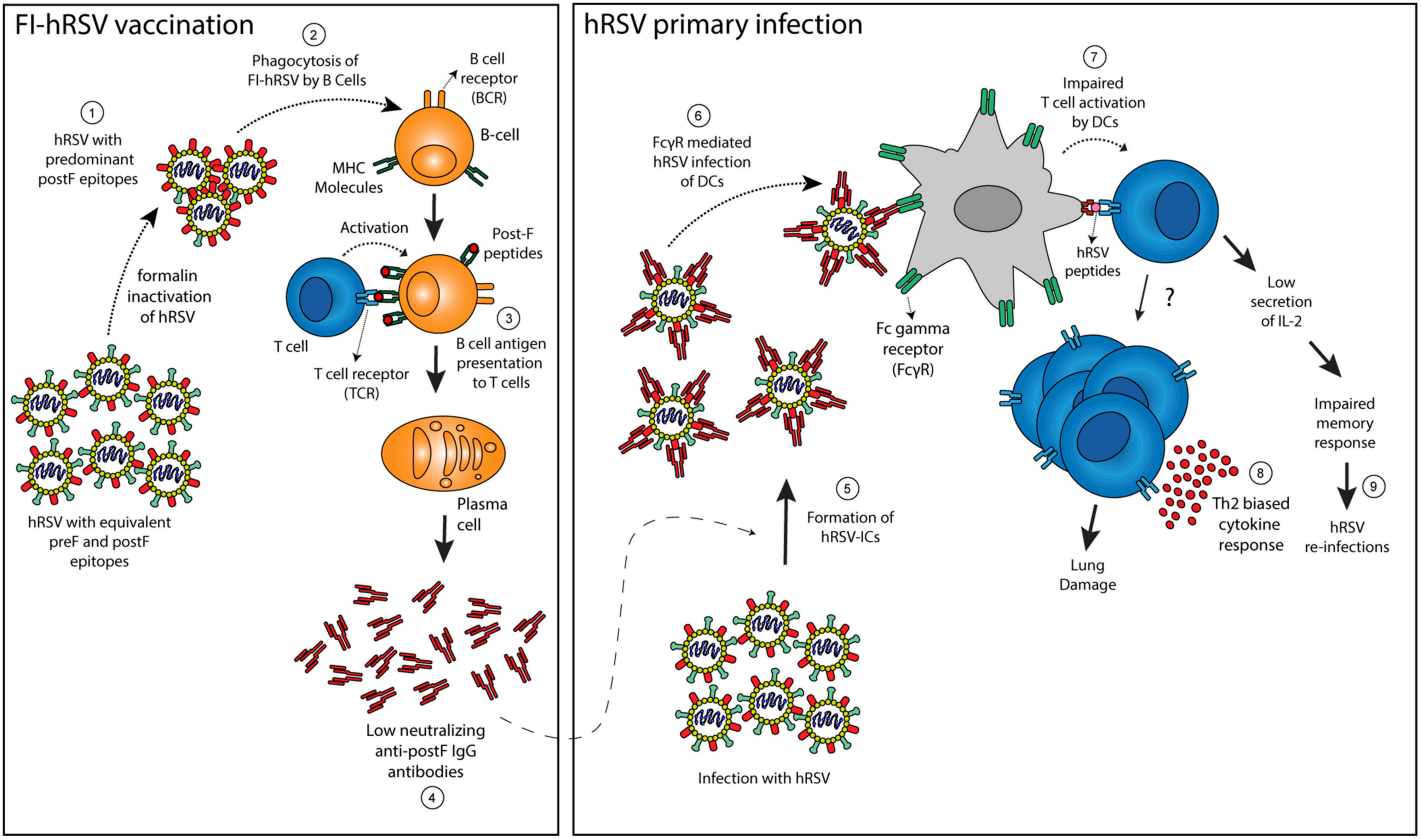
Proposed mechanism to explain enhancement of hRSV-induced disease following FI-hRSV vaccination. Formalin hRSV inactivation produces a non-infectious virus with a high proportion of post-fusion conformation epitopes in the F protein (Post-F) (1). The inactivated virus is then phagocyted by B cells (2) that can present hRSV antigens to T cells in the context of MHC molecules (3). The interaction between B and T cells allows the differentiation of B cells into plasma cells that generate antibodies against the post-fusion conformation of the hRSV F protein (4). Such antibodies failed to neutralize hRSV infection but also may enhance the infection of FcγR bearing cells such as DCs. When infection by hRSV occurs, the low neutralizing antibodies induced by the FI-hRSV vaccine can form immune-complexes (ICs) with hRSV (5) that leads to the activation of Fc-gamma receptors expressed on the surface of DCs (6). Subsequently, an impaired DC-mediated T cell activation (7) can induced CD4^+^ T cells with a Th2-biased phenotype that promotes lung damage (8). Furthermore, low secretion of IL-2 by CD4^+^ cells activated by hRSV-IC-loaded DCs can lead to a poor memory response that contributes to hRSV re-infection (9).

The administration of a formalin-inactivated hRSV vaccine to children nearly 50 years ago, which was aimed at preventing severe respiratory disease elicited by hRSV infection was unable to produce protective immunity against hRSV. Contrarily to what was expected, its administration resulted in increased morbidity and mortality in vaccinated infants when they were later infected by the virus (Kim et al., 1969). Although the mechanisms underlying the pathological effects of FI-RSV vaccine have not been totally elucidated, this episode revealed complexities associated to vaccine development, which has been hampered, and raised hypothesis about the pathologic roles of hRSV-ICs (Kim et al., 1969; Polack et al., 2002; Delgado et al., 2009). Enhanced hRSV disease (ERD) after FI-RSV immunization of BALB/c mice has been associated with alveolar deposition of ICs, which was observed 7 dpi of hRSV by means of co-localization of IgG with the complement component 3 (C3 protein). The role of complement fixing ICs in ERD was supported by experiments in C3^−/−^ mice, which showed significantly less airway hyper-responsiveness (AHR) in comparison to WT counterparts, after FI-hRSV vaccination and hRSV challenge, arguing for a role of complement in bronchoconstriction observed in ERD (Polack et al., 2002). These experimental studies were supported by histological analysis of lung sections from two infants that suffered fatal ERD, in which IC-mediated complement activation was observed through extensive peribronchiolar complement component 4d (C4d) deposition in the airway tissue (Polack et al., 2002). Furthermore, a sub-optimal, non-protective antibody response in mice, characterized by high levels of non-neutralizing anti-F and anti-G IgG antibodies, was observed after immunization with FI-hRSV, but not infectious hRSV (Polack et al., 2002). The lack of affinity maturation in Abs elicited by FI-hRSV was associated with enhanced lung histopathology and AHR, whereas the supplementation of Toll-like receptor (TLR) agonists, performed during immunization promoted proper affinity maturation that prevented ERD after hRSV challenge, showing that a deficient TLR stimulation in B cells is likely responsible for the lack of Ab affinity maturation after FI-hRSV vaccination (Delgado et al., 2009). Furthermore, cotton rats vaccinated with FI-RSV elicited high levels of hRSV-specific antibodies, which displayed low neutralizing titers in Vero cells (Piedra et al., 1993). These antibodies were also able to cause ADE in *in vitro* assays. These studies suggest that sub-optimal antibody production and the generation of ICs play a role in ERD development (Figure 2). Furthermore, recent studies suggest that CD4^+^ subsets and a Th2-biased immune response are key for AHR and ERD (Knudson et al., 2015). In this context, TAM (Tyro3, Axl, and Mertk) receptors, which are expressed in various cells and tissues, and their ligand Growth arrest-specific 6 (Gas6) could be involved in the production of a Th2-biased immune responses that reduce the production of type IgG2a subclass antibodies (Shibata and Ato, 2017). These antibodies could have an effective neutralizing capacity against hRSV and therefore prevent hRSV induced disease, but their production is lowered as a consequence of FI-hRSV immunization followed by hRSV infection. Therefore, it is possible that the TAM/Gas6 signaling axis can contribute to the generation of low neutralizing antibodies that failed to neutralize hRSV infection and instead contributes to the pathology caused by hRSV infection through the engagement of FcγRs.

## Concluding Remarks

The hRSV is a leading cause of respiratory illness in infants and a major health burden worldwide. Re-infections with this virus are common and can contribute to additional clinical manifestations, such as asthma and allergies. For this reason, several studies have focused on understanding the mechanisms that can contribute to hRSV induced pathology, but also to elucidate the factors that contribute to re-infection episodes throughout life. In this context, some studies suggested that low number of memory hRSV-specific CD8^+^ T cells could be associated with re-infection episodes and that the levels of such cells could be regulated by virus-specific antibodies, by modulating the function of antigen presenting cells, such as DCs. Furthermore, recent studies suggest that the generation of regulatory memory T cells could be impaired by the interaction of hRSV-ICs with DCs, pointing out these phenomena as an interesting research topic that deserves analysis. In this review, and based on several studies, we discussed the role of FcγRs during hRSV infection and their immune-modulatory properties that can account for recurrent hRSV infection episodes and the enhancement of the disease caused by FI-hRSV vaccination. However, further research is needed to understand how hRSV induces the production of antibodies that fail to prevent re-infections. Knowledge of such mechanisms would certainly be appreciated for vaccine and therapy development against hRSV, which represents a major global health problem.

## Author Contributions

OA and FD are responsible for the writing of this review article. TB, FB, JE-V, JS, and PG reviewed the manuscript. AK is the leading investigator and assisted in the organization and revision of this article. All authors listed approved the version to be published and have made a substantial and intellectual contribution to the work.

### Conflict of Interest Statement

The authors declare that the research was conducted in the absence of any commercial or financial relationships that could be construed as a potential conflict of interest.
